# Methanol Adsorption and Reaction on Samaria Thin Films on Pt(111)

**DOI:** 10.3390/ma8095302

**Published:** 2015-09-17

**Authors:** Jin-Hao Jhang, Andreas Schaefer, Volkmar Zielasek, Jason F. Weaver, Marcus Bäumer

**Affiliations:** 1 Institute of Applied and Physical Chemistry & Center for Environmental Research and Sustainable Technology, University Bremen, Leobener Strasse UFT, Bremen 28359, Germany; E-Mails: jhang@uni-bremen.de (J.-H.J.); zielasek@uni-bremen.de (V.Z.); mbaeumer@uni-bremen.de (M.B.); 2 Department of Chemical Engineering, University of Florida, Gainesville, FL 32611, USA; E-Mail: jweaver@che.ufl.edu

**Keywords:** rare earth oxide, thin film, samaria, inverse catalysts, temperature programmed desorption (TPD)

## Abstract

We investigated the adsorption and reaction of methanol on continuous and discontinuous films of samarium oxide (SmO*_x_*) grown on Pt(111) in ultrahigh vacuum. The methanol decomposition was studied by temperature programmed desorption (TPD) and infrared reflection absorption spectroscopy (IRRAS), while structural changes of the oxide surface were monitored by low-energy electron diffraction (LEED). Methanol dehydrogenates to adsorbed methoxy species on both the continuous and discontinuous SmO*_x_* films, eventually leading to the desorption of CO and H_2_ which desorbs at temperatures in the range 400–600 K. Small quantities of CO_2_ are also detected mainly on as-prepared Sm_2_O_3_ thin films, but the production of CO_2_ is limited during repeated TPD runs. The discontinuous film exhibits the highest reactivity compared to the continuous film and the Pt(111) substrate. The reactivity of methanol on reduced and reoxidized films was also investigated, revealing how SmO*_x_* structures influence the chemical behavior. Over repeated TPD experiments, a SmO*_x_* structural/chemical equilibrium condition is found which can be approached either from oxidized or reduced films. We also observed hydrogen absence in TPD which indicates that hydrogen is stored either in SmO*_x_* films or as OH groups on the SmO*_x_* surfaces.

## 1. Introduction

Rare earth oxides (REOs), especially the oxides of lanthanum and cerium, find their main applications as catalysts in the chemical industry. Lanthana is mostly employed for so called “fluid cracking” to break up long-chain hydrocarbons, whereas ceria, at times with additives of praseodymia, is employed as an oxygen storage material for exhaust gas cleaning in heterogeneous reactions (e.g., CO oxidation, NO reduction in automotive catalysts) [1]. Basic research has thus mainly focused on ceria and lanthana [2]. Nevertheless the other oxides of the rare earth elements have also shown promising potential in diverse fields like microelectronics [3] or in heterogeneously catalyzed reactions for which the selective formation of an intermediate product has increased importance. As examples, reactions such as the oxidation of organic compounds [4], the oxidative coupling of methane [5,6,7], and the water-gas shift reaction [8,9] can be mentioned.

The versatility of certain rare earth oxides, as for many transition metal oxides, has its origin in the ability of the metal ion to easily switch its oxidation state. All rare earth elements can exist in a trivalent state (3+) and form a sesquioxide, RE_2_O_3_. Only cerium, praseodymium, and terbium can form a dioxide with the metal in the 4+ oxidation state [10]. Also, these oxides form many structural phases with intermediate oxygen contents. In their stable forms under atmospheric conditions CeO_2_, Pr_6_O_11_ and Tb_4_O_7_ have shown superior lattice oxygen mobility and a number of stable intermediate crystallographic phases. This property makes them good catalysts for total oxidation reactions and ceria has been studied extensively also in the field of surface science. Additionally, it has recently been found that inverse model catalyst systems of CeO*_x_* nanoparticles grown for example on Au and Cu substrates exhibit high reactivity for the water-gas shift reaction [9,11,12]. However, only few studies so far attempt to study model systems of REOs other than ceria [13,14,15]. Apart from CeO*_x_*, PrO*_x_* and TbO*_x_*, REOs only form sesquioxides (RE_2_O_3_) which exhibit a relatively low mobility of lattice oxygen. The lower oxygen mobility can be beneficial for the selectivity of certain chemical conversions such as, for example, the oxidative coupling of methane (OCM). In the OCM reaction sesquioxides like La_2_O_3_, Sm_2_O_3_ or Eu_2_O_3_, among other sesquioxides and certain REO mixtures, have shown an increased C2 selectivity [6]. Oxides of Sm or Eu could be of special interest, because they possess the capability to change their oxidation state to 2+, as reported under extreme conditions for the bulk oxides [10]. However, surface science studies of sesquioxide model systems are scarce or lacking completely in the context of model catalysis, and the relationship between surface structure and reactivity of the oxides is still largely unknown.

In a previous study combining scanning tunneling microscopy (STM) and low-energy electron diffraction (LEED) we have demonstrated that a high-quality Sm_2_O_3_(111) thin film can be grown on Pt(111) by reactive physical vapor deposition (RPVD) [13]. Growing a thin oxide film on a conducting substrate allows for employing the whole set of surface science methodology to study structure, stability, and reactivity of the oxide surface. Furthermore structures may be stabilized which are not stable in bulk oxides under atmospheric conditions [16]. If discontinuous films of the oxide are prepared, the influence of the metal-oxide interface at the boundary of the oxide islands to the Pt substrate can be studied and possible cooperative effects of the two materials revealed.

As shown in Ref. [13], samaria grows in a defective fluorite structure on Pt(111), in which the Sm atoms form a well-ordered (1.37 × 1.37) hexagonal sub-lattice in registry with Pt(111), whereas oxygen vacancies are distributed randomly within the film. We found that the Sm_2_O_3_ films can be reduced thermally, forming domains of SmO(001), and re-oxidized by annealing in molecular oxygen. Hence, a reduction re-oxidation cycle is in principle possible on SmO*_x_* thin films. In the present article we report a study of the adsorption of methanol (MeOH) on as-prepared, reduced, and re-oxidized SmO*_x_* surfaces. MeOH is considered as a versatile probe molecule for characterizing chemical properties of metal oxide surfaces. It has been shown that the dissociation and oxidation of MeOH on metal oxides are strongly affected by the nature of metal oxides. The reaction products can indicate the nature of active sites on metal oxide surfaces because Lewis and Brønsted acid sites dehydrate MeOH to form dimethyl ether, redox sites oxidatively dehydrogenate MeOH to form formaldehyde, and basic sites dehydrogenate MeOH to form CO and CO_2_. Studies of MeOH oxidation reaction also have been performed on diverse ceria surfaces, e.g., CeO_2_(111), CeO*_x_*(111), and CeO*_x_*(100) using classical surface science tools [17,18,19]. The results reveal that formaldehyde and H_2_O as products are formed on the CeO_2_(111) surface, whereas CO, CO_2_, and H_2_ are produced from both CeO*_x_*(111) and CeO*_x_*(100) surfaces, suggesting that the chemical behavior of the ceria surface can be altered by its surface structure as well as oxidation states.

In this article we study the adsorption and reaction of MeOH on a continuous film, only a few O-Sm-O trilayers thick, compared to a discontinuous film of SmO*_x_* islands which form at sub-monolayer coverage and leave Pt(111) area exposed. In addition, we investigate the effect of reduction and reoxidation of the oxide film on the reactivity and discuss the influence of the presence of both oxide and metal surface sites in close proximity on the reaction mechanism. By combining temperature-programmed desorption (TPD), infrared reflection-absorption spectroscopy (IRRAS), and LEED with structural information previously determined by STM, we find strong correlations between the SmO*_x_*/Pt(111) film structure and its chemical properties.

## 2. Experimental Section

The experiments were carried out in two UHV chambers each with a base pressure below 5 × 10^−10^ mbar. The first chamber is equipped with a quadrupole mass spectrometer (HIDEN Analytical Ltd., Warrington, UK), an e-beam evaporator (EFM3 by Scienta Omicron, Taunusstein, Germany), an ion sputter source (SPECS GmbH, Berlin, Germany), a four-grid LEED (Scienta Omicron, Taunusstein, Germany), a X-ray gun (Scienta Omicron, Taunusstein, Germany), and a hemispherical electron analyzer (E10 by former Leybold AG, Hanau, Germany). The second chamber houses an IR spectrometer with MCT detector for infrared reflection absorption spectroscopy (IRRAS, Vertex 80v by Bruker Optic GmbH, Ettlingen, Germany) and an e-beam evaporator (EFM3), ion source and a combined LEED/AES optics (SPECS GmbH, Berlin, Germany).

The oxide films were grown on a Pt(111) single crystal (SPL). The crystal is a circular disk (9 mm diameter) which was mounted on a molybdenum sample plate. A type-K thermocouple was spot-welded onto the backside of the Pt(111) crystal for temperature measurement. The Pt(111) crystal surface was cleaned by repeated cycles of Ar^+^ sputtering (1 keV) at room temperature for 20 min with annealing in UHV at 1000 K for 15 min, followed by annealing in O_2_ (P = 5 × 10^−7^ mbar) for 10 min at the same temperature. The surface is considered clean when the C and O signals in X-ray photoelectron spectroscopy (XPS) or Auger electron spectroscopy (AES) were below the detection limits (XPS: atomic ratios of C/Pt and O/Pt < 0.02; AES: atomic ratios of C/Pt and O/Pt < 0.03). 

Samaria thin films were grown by reactive physical vapor deposition (RPVD) in O_2_ background (*P* = 5 × 10^−7^ mbar) at 600 K crystal temperature followed by post-annealing in O_2_ background (*P* = 5 × 10^−7^ mbar) at 1000 K for 10 min. The estimation of the film thickness is based on attenuation of the Pt 4f_7/2_ peak intensity in XPS spectra (Al K_α_ radiation). An inelastic mean free path of 25.03 Å is used for the Pt 4f_7/2_ photoelectrons with the kinetic energy of 1414.7 eV in the XPS/TPD chamber. In the IRRAS chamber, the film thickness is estimated by measuring the attenuation of the Pt (NOO) peak intensity at 168 eV in AES spectra and an inelastic mean free path of 5.53 Å. The IMFP values from NIST database are determined by considering a bulk c-Sm_2_O_3_ (density: 8.347 g/cm^3^) grown on Pt(111). The thickness of one monolayer (ML) of Sm_2_O_3_ (111) is defined as equal to 3.18 Å, which corresponds to the average length of a trilayer O-Sm-O along the <111> direction of a c-Sm_2_O_3_ bixbyite structure. For TPD experiments, 0.9 ± 0.1 ML and 2.8 ± 0.1 ML Sm_2_O_3_ thin films were grown on the Pt(111) substrate. A 0.7 ± 0.2 ML Sm_2_O_3_ thin film was prepared for the IRRAS experiments. A well ordered Sm_2_O_3_ (111) thin film exhibits a hexagonal (1.37 × 1.37) lattice pattern in registry with Pt(111)-(1 × 1) in LEED. In the coverage range of 1 to 3 ML, additional spots can be observed which originates from a quasi-3 × 3 superstructure relative to the Sm_2_O_3_ lattice. For further details see [13]. 

MeOH TPD was performed by dosing 15 Langmuir (L) of MeOH purified by five freeze-pump-thaw cycles onto a sample held at 96 K at a background pressure below 5.0 × 10^−10^ mbar. The temperature was ramped with a well-controlled rate of 1 K/s from 100 to 800 K. The signal of six masses (*m*/*z* = 2 (H_2_), 18 (H_2_O), 28 (CO), 29 (CH_2_O), 31 (CH_3_OH) and 44 (CO_2_)) were monitored. Repeated TPD experiments were performed immediately after a previous TPD experiment when the sample has returned to < 100 K and further MeOH could be dosed. The intensities of recorded masses were normalized to the ionization probability of the respective molecule. To better identify small desorption features some spectra were multiplied by factors given in the respective figure. For the quantification of products, we define one monolayer equivalent (MLE) as the surface atomic density of Pt(111), being 1.52 × 10^15^ cm^−2^. For calibration of the TPD signal intensity 1/3 MLE of CO was dosed to clean Pt(111) at room temperature, forming a (√3 × √3)R30° diffraction pattern in LEED. The integral intensity of the CO desorption signal corresponding to 1/3 MLE is then taken as the reference for all yields determined in this study.

IRRAS results were obtained by guiding infrared light through a KBr window to the sample at an angle of incidence of ~83° to the sample surface normal. The reflected beam passes through another KBr window and impinges onto a nitrogen cooled HgCdTe (MCT) detector. Spectra were recorded with a resolution of 2 cm^−1^ and over 200 scans were averaged between 600–4000 cm^−1^. Additionally, for the IRRAS experiments 15 Langmuir of MeOH were dosed to the sample at 96 K and a background pressure below 5 × 10^−10^ mbar. For the heating series the sample was heated to the respective temperature, held for one minute and then rapidly cooled again to ~96 K before the measurement was started.

## 3. Results and Discussion

### 3.1. MeOH on Clean Pt(111)

**Figure 1:**
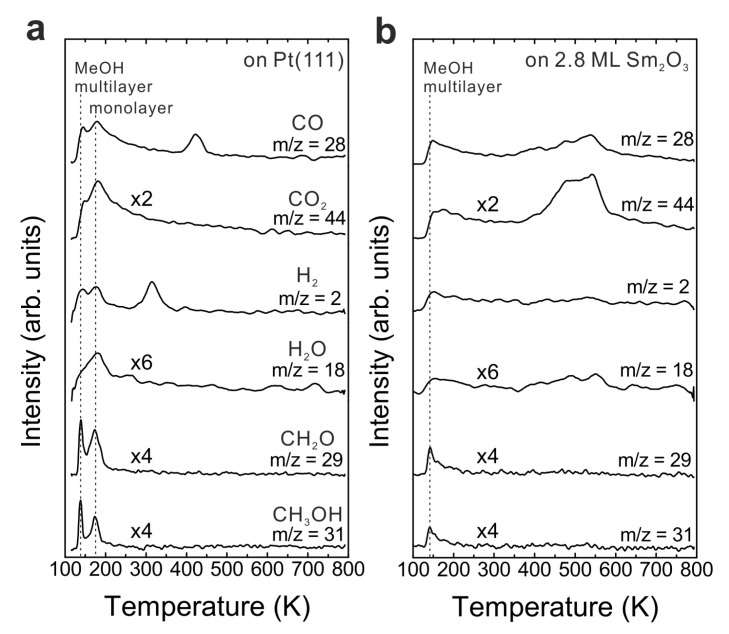
TPD spectra recorded from (**a**) a clean Pt(111) and (**b**) a 2.8 ML as-prepared Sm_2_O_3_ thin film.

To determine the contribution of the Pt(111) substrate to the MeOH reaction, we carried out a MeOH TPD experiment on a clean Pt(111) surface by dosing 15 Langmuir of MeOH on the surface at a substrate temperature of 96 K. The TPD spectrum is shown in Figure 1a. In the TPD spectrum, the multilayer and monolayer desorption of MeOH are observed at 140 and 180 K, respectively. All signals detected below 250 K are caused by fragmentation of MeOH in the mass spectrometer. As products, only H_2_ and CO are observed with peak positions at 315 and 420 K, respectively. The yields of products will be quantified in units of monolayer equivalents (MLE) in the following. One MLE is defined as the surface atomic density of Pt(111), that is, 1.52 × 10^15^ cm^−2^. The integral intensity of the TPD peak of 1/3 MLE CO on Pt(111) was employed for calibration as described in the experimental section. Only small yields of CO (0.04 ± 0.01 MLE) and H_2_ (0.07 ± 0.01 MLE) are produced from the reaction on Pt(111). The average ratio of H_2_ to CO is approximately two, suggesting complete decomposition of the reacting MeOH and desorption of products via CH_3_OH → CO + 2 H_2_. The amount of CO is equal to the amount of reacted MeOH on Pt(111), *i.e.*, only 0.04 ± 0.01 MLE MeOH do react, showing that Pt(111) exhibits minor activity for MeOH decomposition. Our result is in good quantitative agreement with previous MeOH TPD experiments on clean Pt(111) which found a maximum amount of 0.047 MLE MeOH decomposing [20]. Previous studies suggest that defects on the Pt(111) surface play an essential role for promoting the MeOH reaction, and that the intermediates of the MeOH reaction leading to final products (CO and H_2_) are nearly undetectable on Pt(111) experimentally [21,22,23]. Theoretical studies of the MeOH reaction on Pt(111) have been performed as well, but there is still no agreement/conclusion on whether the first step of MeOH reaction on Pt(111) proceeds by the C-H bond scission to form hydroxymethyl or by O-H bond scission to form methoxy [24,25].

### 3.2. As-Prepared Continuous Sm_2_O_3_ Film

Figure 1b shows TPD traces obtained after dosing 15 Langmuir of MeOH on a Sm_2_O_3_ thin film with a nominal thickness of 2.8 ± 0.1 ML at the surface temperature of 96 K. No peaks are observed in the temperature range of H_2_ and CO desorption from MeOH reaction on Pt(111), suggesting that the Pt substrate is fully covered by the Sm_2_O_3_ thin film. Compared to the desorption features from Pt(111), a smaller MeOH multilayer desorption peak appears at the same temperature as from the bare Pt(111) surface. Furthermore, the desorption feature of a MeOH monolayer can be hardly discerned which suggests that most of the adsorbed MeOH monolayer is able to react on the Sm_2_O_3_ surface. Different from the MeOH decomposition on Pt [26], the MeOH reaction on the Sm_2_O_3_ film leads to CO and CO_2_ as the main products detected in the range of 400–600 K in the TPD spectrum. No other products are observed. Quantification reveals that 0.13 ± 0.01 MLE CO and almost the same amount of CO_2_ (0.14 ± 0.01 MLE) are produced. In addition, the MeOH turnover values hence are 0.27 MLE on the continuous Sm_2_O_3_ thin film compared to 0.04 MLE on Pt(111) showing that the Sm_2_O_3_ thin film exhibits much higher reactivity than a bare Pt(111) surface. 

The production of CO_2_ is intriguing because it indicates that there is oxygen from the oxide available for CO_2_ formation. We consider three possible sources for this “active” oxygen contributing to CO_2_ formation: (1) surface OH, (2) lattice oxygen and (3) weakly bound molecular oxygen species on the SmO*_x_* surface. Surface OH could react with CO and form, via carboxyl (HOCO) and/or formate (HCOO) species as intermediates, CO_2_ and H as products [26,27]. The lattice oxygen on the Sm_2_O_3_ surface could contribute by fully oxidizing MeOH to CO_2_, via reduction of the oxide. A similar behavior has been found on lanthana thin films grown on an Al_2_O_3_ (0001) substrate by examining CO oxidation to CO_2_ which, as a result, leads to reduction of the lanthana thin films [28]. Gorte *et al.* demonstrated the existence of weakly bound oxygen on a CeO_2_(111) surface which desorbs in a temperature range between 800 and 1200 K [29]. We checked our Sm_2_O_3_ thin films for such a species by recording an O_2_ TPD spectrum (not shown) between 300–1000 K with a heating rate of 1 K/s, but did not observe O_2_ desorption. Therefore, we conclude that both surface OH and lattice oxygen can be oxygen sources for the CO_2_ formation, while no weakly bound oxygen species is present on the Sm_2_O_3_ thin film.

Quantifying our MeOH TPD result for the closed Sm_2_O_3_ film reveals that a considerable amount of H (1.08 MLE) is missing in the balance, *i.e.*, not desorbing from the surface. Amounts of missing H in TPD suggests that H atoms remain on or in the SmO*_x_*/Pt(111) sample, possibly via OH formation on the SmO*_x_* surface and/or by diffusion into the SmO*_x_* film or even into the Pt substrate. Considering one trilayer (O-Sm-O) in a bulk Sm_2_O_3_ in bixbyite structure, there are only 0.40 MLE top-layer O exposed, meaning that only a maximum of 0.40 MLE H can react with top-layer O forming OH. Consequently, a minimum of 0.68 MLE H must diffuse into the oxide and/or to the Pt-oxide interface, or into the Pt substrate.

**Figure 2:**
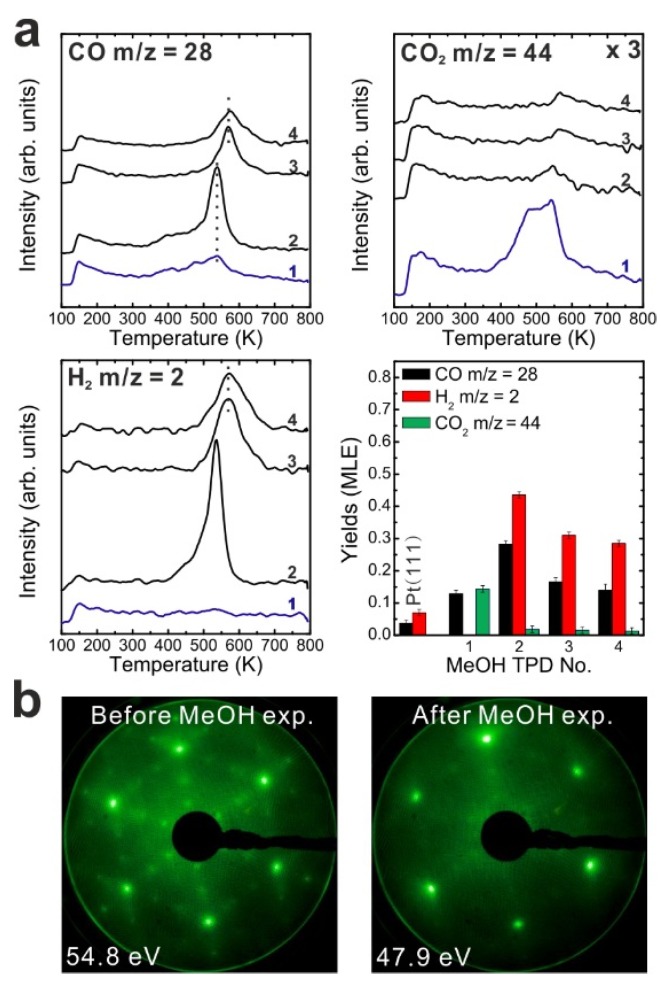
(**a**) Repeated TPD series collected on a 2.8 ML as-prepared Sm_2_O_3_ thin film and the estimated yields of products, (**b**) LEED results obtained before and after the repeated MeOH TPD experiments.

To determine if MeOH adsorption, reaction and desorption of the products alter the chemical properties of the samaria surface, we performed repeated MeOH TPD experiments. The corresponding TPD spectra obtained from the 2.8 ML as-prepared Sm_2_O_3_ thin film are shown in Figure 2a. The second MeOH TPD experiment was carried out immediately after the first TPD run and involved cooling to 96 K and adsorbing 15 Langmuir of MeOH again. The spectra exhibit significant changes in the CO, CO_2_ and H_2_ TPD features. We observe that CO and H_2_ desorb concurrently in large TPD peaks at 540 K. Compared to the 1st TPD results, the CO_2_ desorption peak intensity is lower by a factor of seven and the integrated intensity of the CO peak is higher by a factor of 2.15, while the peak maxima have a ratio of 3.6. The strong decrease of the CO_2_ desorption peak suggests that most of the “active” oxygen was removed from the surface during the 1st TPD run. Afterwards, the third TPD and the fourth TPD were also carried out immediately after their previous TPD run. There is no significant difference between the third and the fourth TPD, however, both of them show that the CO, CO_2_, and H_2_ desorption peaks shift to higher temperature by 30 K compared to the second TPD. Overall, the product yields slightly decrease after the second TPD experiment. The fact that MeOH reacting on the SmO*_x_* thin film forms CO, CO_2_ and H_2_ as the main products suggests that dehydrogenation of MeOH is the dominant reaction. MeOH dehydrogenating to methoxy species (CH_3_O^−^) has been well-studied, especially on metal oxides. Moreover, the methoxy species can further completely dehydrogenate to CO and H_2_ as the final products. Hence, according to our TPD result, SmO*_x_* is a basic oxide as revealed by the MeOH reaction. MeOH reacting on an acidic surface would undergo a dehydration reaction and form dimethyl ether as the final product [30,31] which we do not observe. Furthermore, the TPD result suggests that the C–H bond scission of methoxy species is the rate-determining step because the desorption temperature of the products is identical. As soon as the C–H scission takes place, H_2_ and CO (also CO_2_) are formed and desorb from the surface immediately. 

The decrease of the CO_2_ yield during repeated TPD experiments suggests that surface OH and/or lattice oxygen species consumed for CO oxidation are not replenished and should leave surface defects/vacancies behind which could enhance the binding strength between methoxy species and the SmO*_x_* surface. From this point of view, more surface defects/vacancies are created over repeated TPD runs which may explain why product TPD features change and the peaks shift to higher temperatures, meaning that the chemical behavior of SmO*_x_* is changed by the MeOH reaction. It should be mentioned here, that IRRAS data to be presented later support this interpretation.

A deficit of H in the desorption balance is mainly observed in the first TPD experiment, but also occurs in the subsequent TPD experiments. The amount of missing H dramatically decreases from 1.08 MLE to 0.32 MLE after the first TPD which indicates that adsorbed H forming surface OH or diffusing into the oxide can achieve a saturation concentration which is maintained even after the individual TPD experiments. Therefore, less hydrogen from MeOH decomposition can be adsorbed on or by the oxide and must desorb after the first TPD. Nevertheless, we have to point out that the subsequent TPD spectra still indicate that a small amount of H is missing according to the yield estimation. Some of the H may diffuse into the Pt substrate, some may form the background H_2_ desorption signal during sample annealing to 800 K which is not accounted for by integrating the coherent H_2_ desorption peak for the yield estimation. 

We also conducted LEED experiments before and after the MeOH TPD experiments on the Sm_2_O_3_ thin film to learn if the film structure is affected by the MeOH reaction. Before we carried out TPD experiments on the film, the LEED pattern exhibits Sm_2_O_3_ main spots, weak Pt(111) spots and faint quasi-3 × 3 spots which originate from the coincidence lattice formed at the Sm_2_O_3_(111)-Pt(111) interface, as discussed in the experimental section. After the TPD experiments structural changes are indicated by the diffraction pattern. The quasi-3 × 3 spots become blurry and only the main spots from SmO*_x_*(111) can be observed. Since the quasi-3 × 3 pattern requires sufficiently large coincidence areas of the Sm_2_O_3_(111) and Pt(111) lattices, the disappearance of the quasi-3 × 3 pattern suggests that the surface structure of the 2.8 ML as-prepared Sm_2_O_3_ film changes during the TPD experiments, resulting in a loss of order of the coincidence lattice. This structural change is caused by the MeOH reaction, generating further defects/vacancies in the surface, and not simply by the sample heating during the desorption experiments. A test experiment during which we simply annealed an as-prepared Sm_2_O_3_ thin film from 100 to 800 K in UHV several times with a well-controlled heating rate of 1 K/s resulted in no visible changes of the diffraction pattern.

### 3.3. Reduced and Re-Oxidized Continuous Sm_2_O_3_ Film

In a previous structural study of SmO*_x_* thin films on Pt(111) we showed that reduction/reoxidation treatments can alter their surface structure [13]. To obtain insight into correlations of structural and chemical properties of SmO*_x_* thin films, it is instructive to study if and how reduction and reoxidation affect the film’s chemical properties. Hence, we thermally reduced the 2.8 ML as-prepared Sm_2_O_3_ film after the repeated MeOH TPD experiments by annealing in UHV at 1000 K for 30 min. The same treatment was utilized in our previous work [13] and in studies on ceria thin films [32,33]. Annealing of a Sm_2_O_3_ film to 1000 K in UHV can partially reduce the film and generate SmO (100) domains [13].

XPS was used to characterize the films before and after the annealing. Both O 1s and Sm 3d photoemission peaks (see Figure S1 in the Supplementary Information for the XPS data) shift to higher binding energies by 1.0 eV after the thermal reduction. These shifts cannot only be chemical shifts but represent a band bending effect as reported by other XPS studies [34]. A band bending can occur when the electronic structure of the crystalline film surface or the interface between the film and a metallic substrate changes [35], e.g., caused by defect/vacancy formation or SmO formation located on the Sm_2_O_3_ film or at its interface to the Pt(111) substrate which may produce new electronic surface or interface states within the samaria band gap. It is not possible to deduce from our data the origin of the observed band bending. We take the observed shift in the spectra as an indication for the reduction of the oxide film.

**Figure 3:**
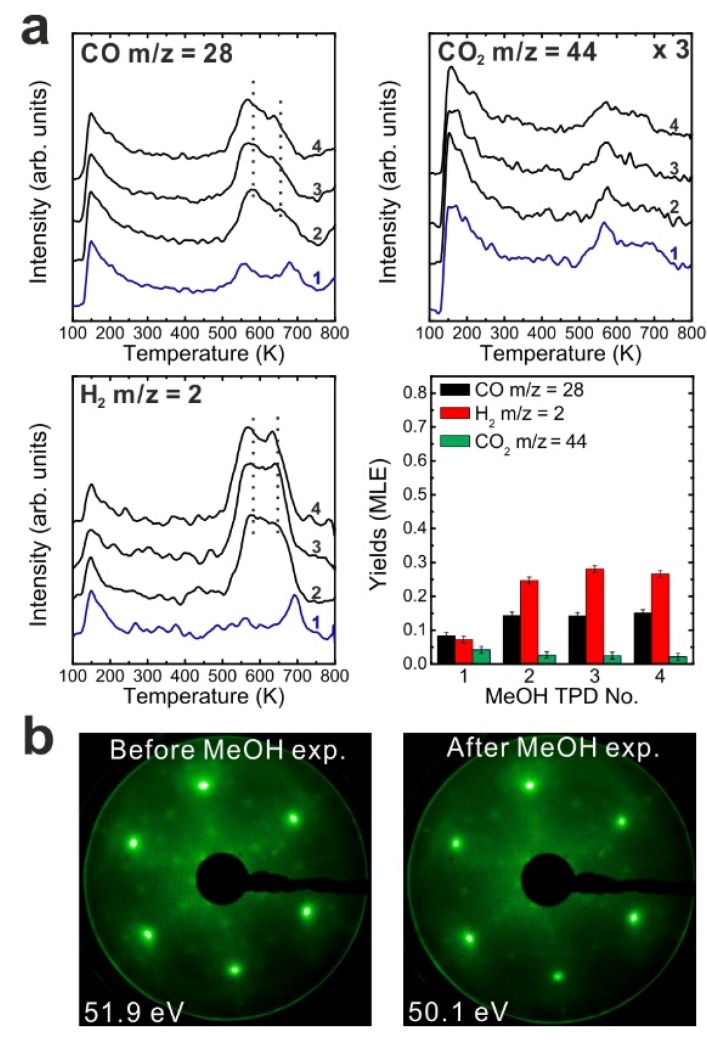
(**a**) Repeated TPD series on a 2.8 ML SmO*_x_* film after a treatment of thermal reduction and the estimated yields of products, and (**b**) LEED results before and after the repeated MeOH TPD experiments.

After the reduction treatment, repeated TPD experiments were conducted on the reduced SmO*_x_* thin film. The results are summarized in Figure 3a. In the first TPD spectrum, relatively small amounts of products are observed compared to the 2.8 ML as-prepared film. Two CO desorption peaks appear at 550 and 680 K as well as a tiny H_2_ peak at 690 K. CO_2_ is also observed in this temperature range. Only 0.12 ± 0.01 MLE MeOH react on the reduced film, which is less than half of the reaction yield observed for the as-prepared film. Again, neither MeOH recombination nor H_2_O or CH_2_O desorption are observed. Less CO_2_ (0.04 ± 0.01 MLE) is produced from the MeOH reaction on the reduced SmO*_x_* film as compared to the as-prepared Sm_2_O_3_ film in the first TPD run, confirming that less “active” oxygen (surface OH and/or lattice oxygen) is available on the reduced film for CO_2_ formation. Reaction on the reduced SmO*_x_* gives rise to new desorption peaks of CO (and CO_2_) at 680 K and H_2_ at 690 K, which appear at higher temperatures than those observed on the as-prepared Sm_2_O_3_ thin film. These new desorption peaks indicate that oxide reduction causes the binding between methoxy and the SmO*_x_* surface to strengthen, most likely due to surface defect/vacancy formation, as we also concluded for the as-prepared Sm_2_O_3_ thin films after repeated TPD runs. This interpretation is supported by IRRAS data discussed below. The changes observed in the MeOH-TPD spectra after reducing the Sm_2_O_3_ film reveal that the MeOH reaction on SmO*_x_* surface is affected strongly by the film conditions.

Compared to the first TPD traces, both the CO and H_2_ features are significantly different in the second TPD spectra. Instead of two CO peaks, a broad feature is observed in the second spectrum. Additionally, a broad H_2_ TPD feature appears in the same temperature range between 500 K and 700 K. The complex structure of the TPD features may reflect desorption of products from several different sites at or close to defects/vacancies in the oxide surface. The CO_2_ TPD feature does not change much during the second TPD experiment, only its intensity slightly decreases. During further TPD runs, the features of CO, CO_2_ and H_2_ desorption peaks do not change significantly. At least two peaks of each product are observed and both peaks slightly downshift to lower temperatures by 15 K from the second TPD to the fourth TPD. The yields of CO and H_2_ increase after the first TPD, but remain constant after the second TPD run, whereas the yield of CO_2_ continuously decreases after the first TPD. Similar behavior was also observed on the as-prepared Sm_2_O_3_ thin film as discussed above. Although the CO_2_ yield decreases after the first TPD, a minimum amount of CO_2_ is always detectable in TPD spectra recorded from SmO*_x_* surfaces, indicating replenishment of minor amounts of “active” oxygen after each TPD experiment which may be linked to the reaction of CO with surface OH groups. During sample cooling to 96 K before each subsequent TPD experiment, a small amount of background H_2_O is probably adsorbed forming OH on the SmO*_x_* surface, *i.e.*, the background pressure of H_2_O may provide a minimum amount of OH on the SmO*_x_* surface available for producing CO_2_. 

LEED results obtained from the reduced thin film are shown in Figure 3b. Before TPD experiments, the LEED pattern of the reduced SmO*_x_* film exhibits SmO*_x_* main spots, quasi-3 × 3 spots, and (2√3 × 2√3)R30° spots. The (2√3 × 2√3)R30° pattern was not observed in our previous study, and may correspond to ordered oxygen vacancies or yet unidentified SmO*_x_* structures. We are not able to make a clear assignment at this stage and further structural studies are necessary. After TPD experiments there is no significant change in the LEED pattern, in contrast to what we observed with the as-prepared Sm_2_O_3_ film, suggesting that the long range order of the reduced SmO*_x_* film is not significantly affected by the MeOH reaction. However, we cannot exclude that the MeOH reaction induces some structural change at the surface of reduced SmO*_x_* films, considering that the TPD spectra show slight changes over the repeated TPD experiments.

**Figure 4:**
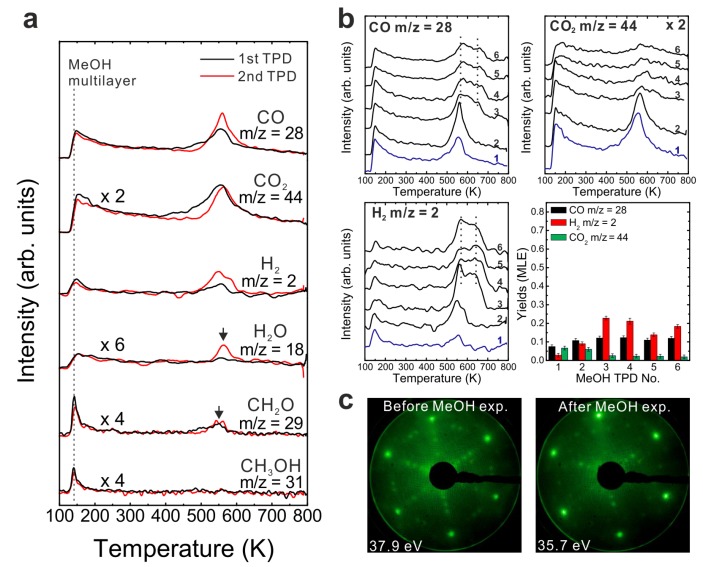
(**a**) The first and second TPD spectra collected from a 2.8 ML SmO*_x_* thin film after the treatment of reoxidation, and (**b**) the repeated TPD series collected on the same sample and the estimated yields of products, and (**c**) LEED results before and after the repeated MeOH TPD experiments.

Subsequent to the reduction treatment of the as-prepared Sm_2_O_3_ film and the TPD experiments performed thereafter, we carried out a reoxidation treatment in order to determine whether a full reduction-reoxidation cycle can restore the chemical activity of the as-prepared film. As shown in our previous study, the reduced SmO*_x_* film can be reoxidized by thermal annealing in molecular oxygen [13]. Here reoxidation of the 2.8 ML reduced SmO*_x_* film was performed by annealing the film in an O_2_ background (P = 5 × 10^−7^ mbar) at 1000 K for 10 min. After annealing in oxygen, both Sm 3d and O 1s peaks in XPS spectra (shown in Figure S1) shifted back by 1.0 eV toward lower binding energies indicating reoxidation. Figure 4a shows the first and second MeOH TPD spectra of the reoxidized film. Surprisingly, small amounts of CH_2_O and H_2_O are observed as products. Also CO, CO_2_ and H_2_ are observed with an identical desorption maximum at 550 K. 

Figure 4b shows TPD spectra collected from repeated MeOH TPD experiments. After the first TPD experiment, the intensities of CO and H_2_ peaks increase, whereas the intensity of the CO_2_ peak slightly decreases in the second TPD spectrum. After the second TPD experiment, no CH_2_O and H_2_O can be detected. The CO, CO_2_ and H_2_ TPD features are similar to the TPD features which we observed from the reduced film, showing a broad feature between 550 and 700 K in the rest of TPD spectra. These results indicate that the reoxidation process does not restore the chemical behavior of the 2.8 ML as-prepared film but generates some oxygen species on or in the film which specifically promotes CH_2_O and H_2_O formation during the first and second TPD experiments. The CH_2_O formation suggests incomplete MeOH dehydrogenation. Moreover, the formation of formaldehyde requires both, weak acidic and basic sites on the reoxidized SmO*_x_* surface in order to limit C-H bond breaking and prevent strong adsorption of CH_2_O on the surface. We, therefore, propose two possible explanations for this observation: first, a special oxygen species such as, e.g., superoxide (O_2_^−^) may form on the SmO*_x_* surface by reoxidation. Such oxygen species have been found and identified on other oxides. For instance, it has been investigated for CeO*_x_* by electron spin resonance (ESR), Raman, and IR spectroscopy [36,37,38]. These oxygen species act as weaker basic sites than lattice oxygen for nearby methoxy species and might be able to break one C-H bond but not promote further dehydrogenation. Instead, these oxygen species might form OH groups reacting with adjacent OH and leaving the oxide surface as H_2_O at the same desorption temperature as formaldehyde. Second, the reoxidation process could improve the flatness of the film resulting in less surface defects, meaning an increase of coordinating numbers of both Sm cations and O anions. Ideally, the CO formation requires four available undercoordinated O anions to dehydrogenate one MeOH molecule via OH formation. An increase of the coordination number of a large fraction of surface O anions could therefore lead to incomplete MeOH dehydrogenation to CH_2_O [18,19].

Overall, the variation of results observed by TPD for as-prepared, reduced, and reoxidized SmO*_x_* films indicates that the chemical properties of the SmO*_x_* surface can be tuned by reduction and oxidation procedures. 

LEED results of the reoxidized film before and after MeOH TPD are shown in Figure 4c. Before the TPD experiments, the LEED pattern of the reoxidized SmO*_x_* film shows Sm_2_O_3_ main spots, weak Pt spots, quasi-3 × 3 spots, and additional 2 × 2 spots with respect to the Sm_2_O_3_ main spots. The additional 2 × 2 spots confirm that the surface structure of the as-prepared Sm_2_O_3_ film is not perfectly restored by the reoxidation treatment. The origin of the 2 × 2 superstructure may be a regular pattern of the proposed special oxygen species located at certain lattice positions or certain oxygen-vacancy ordering but requires further investigation. Over the TPD experiments, the same pattern consisting of SmO*_x_* main spots + quasi-3 × 3 and (2√3 × 2√3)R30° spots was found for the reduced 2.8 ML SmO*_x_* film both before and after repeated MeOH TPD experiments. It strongly suggests that, after repeated MeOH TPD runs, the reoxidized film ends up at a similar structural condition as the reduced film, which is consistent with what we observe in TPD—reactions on the reoxidized film gives rise to similar TPD features as observed from the reduced film after the second TPD experiment. Overall, both LEED and TPD results suggest that the reoxidation indeed generates oxygen species on/in the 2.8 ML reduced film but does not restore the film to the exact same structure and chemical behavior as the as-prepared one. 

### 3.4. As-Prepared Sm_2_O_3_ Islands on Pt(111)

**Figure 5:**
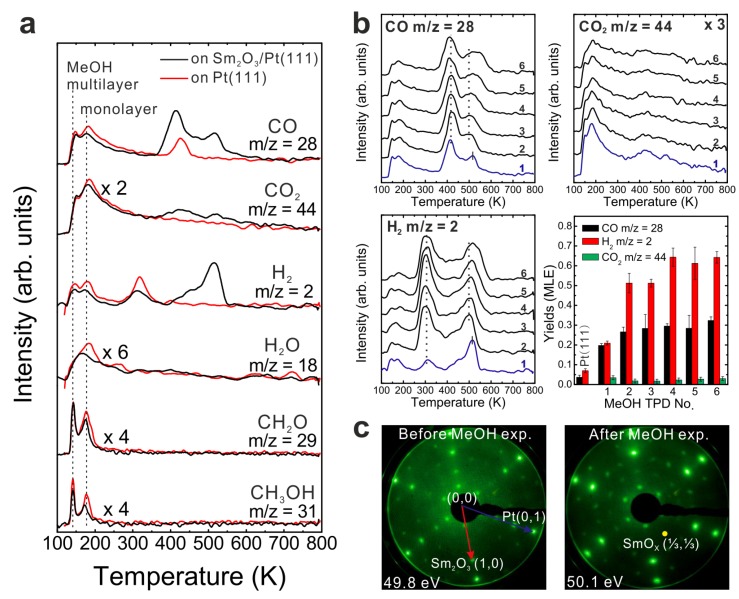
(**a**) The first TPD spectra collected from a clean Pt(111) surface and a 0.9 ML as-prepared Sm_2_O_3_/Pt(111) surface, (**b**) repeated TPD series collected on the as-prepared Sm_2_O_3_/Pt(111) surface and the estimated yields of products, and (**c**) LEED results before and after the repeated MeOH TPD experiments.

MeOH TPD spectra obtained from an as-prepared 0.9 (± 0.1) ML Sm_2_O_3_ film on Pt(111) shows, in contrast to the 2.8 ML SmO*_x_* deposit, both MeOH multilayer and monolayer desorption at 140 and 180 K, respectively (Figure 5a). The presence of a strong MeOH monolayer signal indicates that the Pt(111) surface is not fully covered by the Sm_2_O_3_ thin film because the molecular desorption of MeOH from a monolayer is expected mainly for Pt(111). By estimating the yield of the MeOH monolayer we determine that only ~50% of the Pt(111) surface is covered by the as-prepared Sm_2_O_3_ thin film. More details regarding this estimation will be discussed below in Section 3.6. Also, the TPD spectra exhibit both the H_2_ peak and the CO peak typical for a Pt(111) surface, providing further evidence that there are uncovered Pt areas. Compared to the 1st TPD spectrum obtained from clean Pt(111), the H_2_ TPD peak at 315 K is smaller whereas the CO peak at 420 K is larger for the 0.9 ML Sm_2_O_3_ deposit on Pt(111). In the following, these two peaks are named the Pt-related H_2_ peak and the Pt-related CO peak, respectively. The corresponding H_2_ and CO yields are 0.05 ± 0.01 MLE and 0.12 ± 0.01 MLE, respectively. In comparison to the yields obtained during MeOH TPD on a clean Pt(111) surface (0.07 ± 0.01 MLE of H_2_ and 0.04 ± 0.01 MLE of CO, as mentioned above), we observe three times more Pt-related CO produced on Pt(111) partially covered by Sm_2_O_3_, suggesting a cooperative-effect between SmO*_x_* and Pt, possibly via adsorption sites at the boundary of the SmO*_x_* islands at the interface to the Pt substrate. The amount of Pt-related CO suggests that 0.24 MLE H_2_ should be produced in the balance, but we only observed 0.05 MLE Pt-related H_2_ which indicates that a considerable amount of H remains on and/or diffuses into the sample at this temperature.

The H_2_ and CO desorption peaks that appear at higher temperatures (450–600 K) have no analog in MeOH TPD spectra obtained from bare Pt(111) and must, therefore, be contributions from the SmO*_x_* surface. Both the SmO*_x_*-related H_2_ peak and the CO peak are relatively broad TPD features with an identical position of their maximum at 515 K. Additionally, there is a broad H_2_ feature in the region of 400–450 K which is not observed in the case of the 2.8 ML SmO*_x_* thin film. Its desorption maximum is at a temperature close to that of the Pt-related CO peak. Since we do not observe this H_2_ peak in the MeOH TPD spectra collected from clean Pt(111), we conclude that this H_2_ peak is contributed by the SmO*_x_*-Pt interface at the surface, *i.e.*, the boundary of SmO*_x_* islands where adjacent SmO*_x_* and Pt surface sites exist. As observed in TPD results from the 2.8 ML film, H missing in the balance is also seen for the SmO*_x_*-related H_2_ and CO peaks on the 0.9 ML as-prepared Sm_2_O_3_/Pt(111) system, especially in the first TPD experiment suggesting, as discussed before, OH formation at the surface and/or H diffusion into the sample. According to the yield estimation from both first TPD results, there is a total deficit (sum over Pt- and SmO*_x_*-related TPD peaks) of 0.54 MLE H in desorption from the 0.9 ML Sm_2_O_3_ film, which is half the amount of the missing H in the balance of MeOH TPD on the continuous 2.8 ML Sm_2_O_3_ film, in good correlation with the ratio of the respective surface area covered by Sm_2_O_3_. The result suggests that the Pt(111) substrate does not play a predominant role for storing the missing H. A relatively small CO_2_ peak appears as a broad feature in the temperature range of 350–600 K in the MeOH TPD spectra obtained from the 0.9 ML as-prepared Sm_2_O_3_ film. Compared to 0.14 MLE of CO_2_ produced from the as-prepared continuous Sm_2_O_3_ film in the first TPD, only 0.04 MLE of CO_2_ are produced on the SmO*_x_* islands on Pt(111), meaning that only ~30% of the amount of “active” oxygen on the continuous film must be available on the discontinuous film, in good accordance with the ratio of the total Sm_2_O_3_ coverages of both samples (0.9:2.8).

The results of repeated MeOH TPD experiments on the 0.9 ML as-prepared film are shown in Figure 5b. There is a clear difference in TPD features between the 1st and the subsequent TPD spectra. After the 1st TPD experiment, the intensity of the Pt-related H_2_ peak increases dramatically and the intensity of the Pt-related CO peak increases slightly as well, whereas the intensity of the CO_2_ peak decreases. Now, both the Pt-related CO and H_2_ peaks exhibit much higher intensities than those observed from bare Pt(111). The result suggests that a fraction of the methoxy species adsorbed on SmO*_x_* dehydrogenate to H and CO which spill over to the exposed Pt surface below 300 K and afterwards desorb from Pt as H_2_ and CO at temperatures near 300 and 400 K, respectively. Another fraction of the methoxy species, however, remains on the SmO*_x_* surface until, eventually, producing the SmO*_x_*-related H_2_ and CO TPD peaks at higher temperatures. This interpretation is supported by our IRRAS results in the following section. The Pt-related and SmO*_x_*-related TPD peaks behave differently during repeated TPD experiments. The SmO*_x_*-related H_2_ and CO peaks become broader and shift to higher temperature by 30 K from the 2nd TPD to the sixth TPD experiment, as was also observed for the 2.8 ML film, whereas the Pt-related H_2_ and CO peaks remain at the same temperature without significant changes of their features. These different characteristics reflect that the SmO*_x_* film is altered during repeated methoxy dehydrogenation while the Pt surface remains unchanged.

From the yield estimation we learn that the reactivity of the 0.9 ML film slightly increases after the 1st TPD experiment. The H_2_ yield increases by more than a factor of two and the CO yield also slightly increases after the first TPD experiment by a factor of 1.3, and the average ratio of [H_2_]/{[CO]+[CO_2_]} is estimated as 1.9. Background H_2_ desorption during sample heating to 800 K during TPD might account for the small amount of missing H in the balance calculation, as we discussed before. The yield estimation shows that after repeated TPD runs, the Sm_2_O_3_ islands on Pt(111) also exhibit much higher reactivity for MeOH decomposition than the continuous SmO*_x_* film or the bare Pt(111) surface. The average yield of decomposed MeOH is 0.31 ± 0.03 MLE compared to 0.21 ± 0.01 MLE from the 2.8 ML film and 0.04 ± 0.01 MLE from the the Pt(111) surface, respectively. The continuously higher overall reactivity of the 0.9 ML Sm_2_O_3_/Pt(111) film implies that the cooperative effect between SmO*_x_* and Pt is robust with respect to the changes that repeated MeOH decomposition induces in the SmO*_x_* islands.

The LEED results shown in Figure 5c provide evidence that the 0.9 ML Sm_2_O_3_ layer undergoes structural changes during the repeated MeOH TPD experiments. Before the TPD experiments, the LEED pattern of the sample with SmO*_x_* islands on Pt(111) exhibits sharp and clear Sm_2_O_3_ main spots, Pt spots, and quasi-3 × 3 spots. After the TPD experiments, the latter are slightly rotated and additional spots are observed. One of these additional spots is labeled in Figure 5c, and is located at the (1/3, 1/3) position in registry with Sm_2_O_3_ main spots. Apparently, SmO*_x_* domains slightly rotate against the Pt substrate during the repeated TPD experiments. Moreover, the appearance of the additional spots suggests that the Sm-Sm lattice constant slightly decreases, possibly due to surface defects/vacancies formation and, therefore, the quasi-3 × 3 structure transforms into a (3 × 3) structure. In summary, the film composed of Sm_2_O_3_ islands on Pt(111) exhibits a higher reactivity than both the continuous Sm_2_O_3_ thin film and the bare Pt(111) surface. From the TPD results, the contributions of both Pt and SmO*_x_* surface areas to the MeOH reaction can be identified by monitoring the Pt-related and the SmO*_x_*-related TPD peaks. The SmO*_x_* surface properties are changed by the MeOH reaction for the 0.9 ML and the 2.8 ML thick films. In contrast, the Pt substrate is not affected, as the behavior of SmO*_x_*- and Pt-related TPD features during repeated experiments shows.

**Figure 6:**
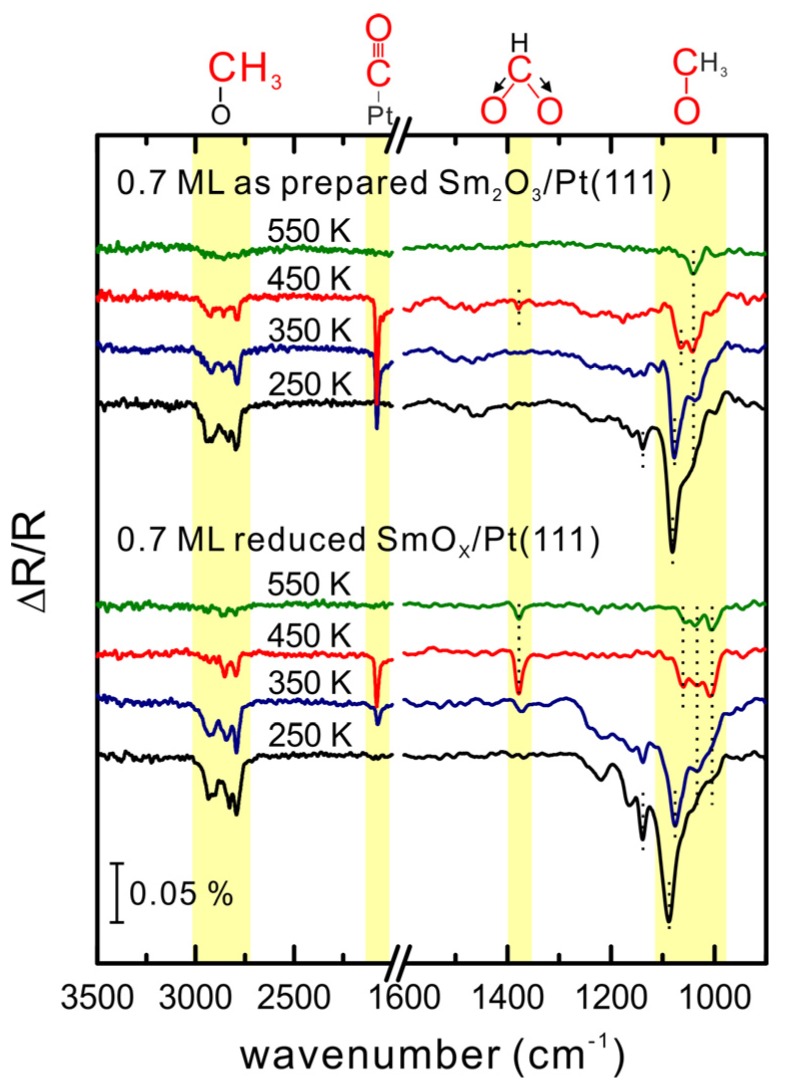
Temperature-dependent IRRAS spectra collected after dosing 15 Langmuir MeOH on a 0.7 ML as-prepared Sm_2_O_3_/Pt(111) and its thermally-reduced SmO*_x_*/Pt(111) surfaces at 96 K and subsequently annealed to particular temperatures.

To extend the understanding of MeOH adsorption and reaction on the SmO*_x_*/Pt(111) system, we also performed IRRAS studies in a different UHV chamber from that in which the TPD studies were conducted (as mentioned in the experimental section). For the IRRAS studies, we prepared a 0.7 ± 0.2 ML Sm_2_O_3_ thin film on Pt(111) and observe the same LEED pattern as that obtained from the 0.90 ± 0.01 ML Sm_2_O_3_/Pt(111) surface used in TPD experiments. Therefore, we consider the film structure of these two samples as comparable. For the IRRAS experiments, 15 Langmuir of MeOH was dosed on the 0.7 ML as-prepared Sm_2_O_3_/Pt(111) surface at 96 K. The elaborate procedure necessary to collect reliable temperature-dependent IRRAS data in our experiments (detailed in the experimental section) made it necessary to restrict the analysis to nearly static conditions of the SmO*_x_*/Pt(111) surface. Therefore, the temperature-dependent IRRAS spectra shown in Figure 6 were collected from the second MeOH reaction on the samples, because from the repeated TPD experiments we found that SmO*_x_*/Pt(111) surfaces show an almost static reactivity after the first MeOH TPD experiment. The IRRAS spectrum collected at 250 K reveals the existence of methoxy species (CH_3_O^−^) which is confirmed by the detection of *v*(C-O), *v*(CH_3_), and ρ(CH_3_) features. The *v*(C-O) peak is broad in the range between 1100 and 1000 cm^−1^, and both *v*_s_(CH_3_) and *v*_as_(CH_3_) peaks are observed at 2790 and 2926 cm^−1^, respectively. Moreover, the ρ(CH_3_) peak is evident at 1140 cm^−1^. The absence of a *v*(OH) signal at 3280 cm^−1^ implies that the O-H bond of methanol breaks. The *v*(CH_3_) frequency is relatively low (*ca.* 2800 cm^−^^1^) from which a low electronegativity for the cationic site can be inferred, indicating that species resulting from the MeOH dehydrogenation are methoxy (CH_3_O^−^) located on top of Sm cations [39,40]. On the basis of TPD results, we speculated that a fraction of the H atoms from the MeOH dehydrogenation diffuses into the film or remains on the surface and forms surface hydroxide. The IRRAS results do not provide evidence for surface OH. It should be noted, however, that because of the small dynamic dipole moment and the adsorption geometry, surface OH on SmO*_x_* is difficult to detect [38]. According to previous studies of MeOH adsorption and reaction on Pt(111) [20], methoxy species are not observed on a bare Pt(111) surface. Methoxy species can exist on an O-terminated Pt(111) surface on which they decompose at temperatures below 150 K. Therefore, we relate all methoxy species detected in the IRRAS spectra above 250 K to Sm_2_O_3_ surface sites.

After heating the sample to 250 K, two *v*(C-O) bands are identified as a sharp peak at 1080 cm^−1^ and a shoulder at 1042 cm^−1^, revealing different adsorption sites for methoxy which can be identified by monitoring the *v*(C-O) frequencies as has been demonstrated in several studies [36,38,39]. If the coordination number of the oxygen atom in the methoxy group is increased from one (on-top site), to two (bridge site) and three (hollow site), the effective mass of this oxygen atom increases as well which leads to a decrease of the observed vibrational frequency for *v*(C-O). Therefore we speculate that the *v*(C-O) band at 1080 cm^−1^ corresponds to the type-I methoxy binding on one Sm cation (on-top), and the broad *v*(C-O) shoulder at lower frequency corresponds to the type-II and III methoxy species bound to two and three Sm cations (bridge and hollow sites). Since this speculation is only based on comparing our IRRAS results with previous IR studies of methoxy on ceria surfaces, theoretical and computational work for MeOH on samaria will be required for confirmation. Most likely, the binding strength between methoxy and the substrate varies for different adsorption sites on the SmO*_x_* surface so that a multitude of different adsorption sites is expected to lead to a broad range of desorption temperatures of SmO*_x_*-related products in TPD spectra.

After heating to 350 K, both the *v*(C-O) and *v*(CH_3_) bands become smaller and the higher frequency *v*(C-O) feature slightly shifts from 1080 to 1077 cm^−1^ which may be due to a dilution effect whereas the lower frequency *v*(C-O) band remains at 1042 cm^−1^. The *v*(C≡O) band observed at 2080 cm^−1^ originates from CO adsorbed on Pt(111). Comparing to TPD results, which showed Pt-related H_2_ desorbing in the temperature range 250–350 K, the IR data reveal that the on-top methoxy species (*v*(C-O) at 1080 cm^−1^) on SmO*_x_* is the source for CO and H_2_ formation on Pt(111) because the *v*(C-O) band at 1077–1080 cm^−1^ has almost vanished at 350 K while the *v*(C≡O) band at 2080 cm^−1^ has intensified. Obviously, the on-top methoxy species dehydrogenates near 250 K to H and CO, which spillover onto Pt in the temperature range 250–350 K. Hydrogen desorbs as H_2_ from the Pt surface which we found in TPD as the Pt-related H_2_ peak, whereas CO remains on the Pt surface in this temperature range.

In the spectrum obtained after sample heating to 450 K, the *v*(C-O) band at 1077 cm^−1^ (on-top methoxy species) has vanished completely. Instead, a *v*(C-O) peak at 1065 cm^−1^ is observed while the *v*(C-O) band at 1042 cm^−1^ remains unshifted. As mentioned before, we speculate that the *v*(C-O) peak at 1065 cm^−1^ and the peak at 1042 cm^−1^ correspond to type-II and III methoxy species on the bridge and hollow sites, respectively. Since the *v*(CH_3_) band has become smaller, less methoxy is present on the surface after heating to 450 K compared with 350 K. While the *v*(C≡O) band is still observed after heating to 450 K, a tiny *v*_s_(OCO) band appears at 1378 cm^−1^, the appearance of which suggests the existence of formate species on the Sm_2_O_3_ surface. Since the *v*_as_(OCO) band expected at ~1550 cm^−1^ is not detected, the formate species must bind to SmO*_x_* as bidentate (C_2v_ and C_s_(1)). The *v*_as_(OCO) of the bidentate species is not IR active due to the metal surface selection rule (MSSR) [41,42]. The appearance of formate species and CO can be connected to the transformation of methoxy species on the SmO*_x_* surface. In summary, the decrease of intensity of the *v*(C-O) band, its downshift to 1077 cm^−1^ and disappearance at 450 K indicate turnover of the type-I methoxy species (on-top mode) to CO and H adsorbed on exposed Pt surface, as discussed before, and its transformation to formate on the SmO*_x_* surface.

After the heating step to 550 K, only one small *v*(C-O) band at 1042 cm^−1^ is detected which, in our interpretation, reveals that only a small amount of the type-III methoxy has remained on the SmO*_x_* surface. It should be noted, however, that no sign of the *v*(CH_3_) band is detected which we ascribe to limited sensitivity of IRRAS. In accordance, the TPD spectra show that above 550 K desorption of products is very limited (*cf.* Figure 5). In conclusion, the combined TPD and IRRAS results for MeOH interaction with SmO*_x_* islands on Pt(111) show that the type-I methoxy species (on-top samarium) on the Sm_2_O_3_ surface dehydrogenates at temperatures above 250 K via two routes: (1) transformation to formate on the SmO*_x_* surface. The formate species may be an intermediate and finally decompose to CO_2_ upon further dehydrogenation or to CO while generating OH on the SmO*_x_* surface. (2) Dehydrogenation to CO. The products H and CO spill over to the exposed Pt surface and lead to Pt-related H_2_ and CO peaks in TPD at 300 and 400 K, respectively. Between 350 and 450 K, the type-I methoxy species fully dehydrogenates. The type-II and III methoxy species on the bridge and hollow sites on the Sm_2_O_3_ surface further dehydrogenate only at higher temperature above 450 K and contribute the SmO*_x_*-related H_2_ and CO peaks in TPD. The Pt-related H_2_ and CO peaks are only contributed by the type-I methoxy species which exhibit less stability on the SmO*_x_* surface compared to type-II and III methoxy species.

### 3.5. Reduced and Reoxidized SmO_x_ Islands on Pt(111)

The reduction and reoxidation treatment that was detailed before for the continuous Sm_2_O_3_ film on Pt(111), was also carried out with the SmO_x_ islands on Pt(111). IRRAS results collected from the reduced 0.7 ML SmO*_x_* film on Pt(111) after heating the sample to 250 K (Figure 6) reveal a significant difference in the region of the *v*(C-O) bands compared to IRRAS from the as-prepared film. A broad absorption feature in the *v*(C-O) region appears for the reduced film, and the main *v*(C-O) band which we ascribe to the type-I methoxy species is observed at 1089 cm^−1^ instead of 1080 cm^−1^, *i.e.*, shifted to higher frequency. As for the as-prepared film, the type-I methoxy band is smaller and down-shifted to 1077 cm^−1^, after heating the reduced 0.7 ML SmO*_x_* film to 350 K. Concomitantly, the *v*(C≡O) band at 2080 cm^−1^ as fingerprint for CO on Pt has appeared and suggests, as discussed for the as-prepared film, that H and CO produced from decomposition of type-I methoxy at above 250 K can spill over to the exposed Pt(111). 

After heating the sample to 450 K, the *v*(C-O) band at 1089–1077 cm^−1^ has completely vanished. As for the 0.7 ML as-prepared Sm_2_O_3_ film, both *v*(C-O) fingerprints for type II and type III methoxy species are also observed in the IRRAS spectra from the reduced film, although downshifted by 5 cm^−1^ to 1061 and 1037 cm^−1^, respectively. In addition, a new *v*(C-O) peak at 1005 cm^−1^ is observed which indicates a new adsorption site on the reduced surface for methoxy species (type IV). After heating the reduced film to 550 K, all three *v*(C-O) bands at 1061, 1037 and 1005 cm^−1^ as well as a *v*_s_(OCO) band are small, but still detectable, which is in contrast to the lack of bands observed for the as-prepared film. This result suggests a stronger bonding of methoxy and formate species on the reduced film, probably because more defects/vacancies on the reduced surface are present and assist in stabilizing methoxy and formate species. 

**Figure 7:**
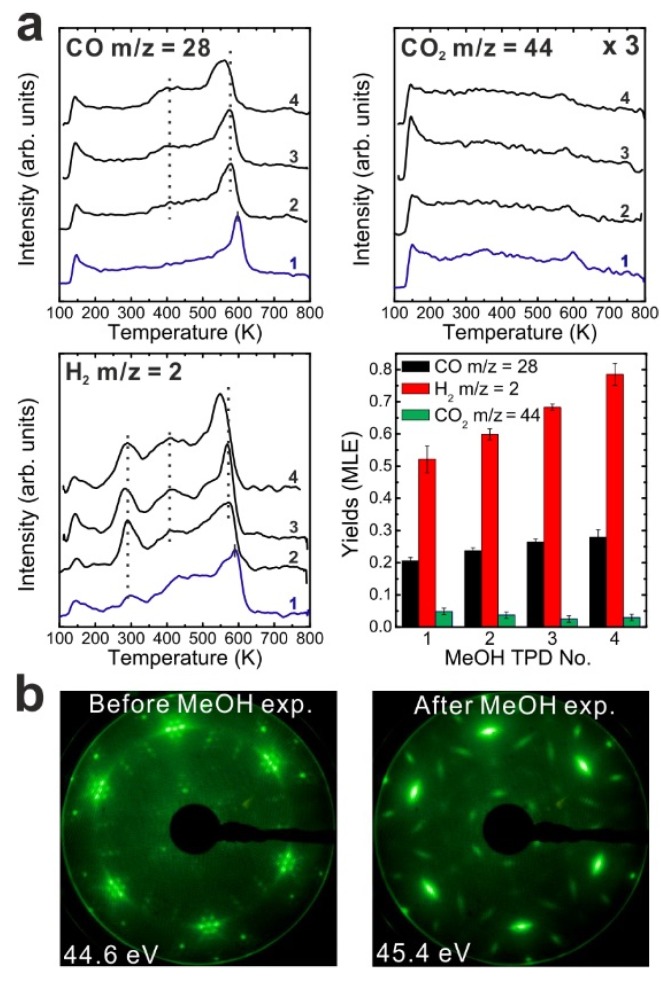
(**a**) Repeated TPD series collected on a 0.9 ML SmO*_x_*/Pt(111) surface after the treatment of thermal reduction and the estimated yields of products, and (**b**) LEED results before and after the repeated MeOH TPD experiments*.*

We also performed repeated MeOH TPD experiments on the reduced 0.9 ML SmO*_x_*/Pt(111) system. Reduction of the oxide was verified by XPS as indicated by shifts of the Sm 3d and O 1s peaks to higher binding energies (shown in Figure S2) that we attribute to a band bending effect as observed on the 2.8 ML reduced film. The TPD spectra recorded from the reduced SmO*_x_*/Pt(111) shown in Figure 7a significantly differ from the ones recorded for the as-prepared film. The Pt-related CO and H_2_ peaks can be hardly discerned in the 1st TPD run. Our previous STM studies demonstrated that upper Sm_2_O_3_ layers from the samaria islands spread onto bare Pt areas during the thermal treatment of the sample for reduction. As a result, less Pt surface is exposed as indicated by the smaller amount of Pt-related CO and H_2_ desorption. While small, the Pt-related H_2_ and CO TPD signals have not shifted with respect to temperature after thermal reduction of the oxide film, even after repeated TPS experiments. This finding is consistent with the assumption based on the IRRAS experiments that the on-top methoxy species dehydrogenates to H and CO which spill over to the exposed Pt surface and desorb from there as H_2_ and CO at 300 and 400 K, respectively.

One clear H_2_ peak appears near 400 K, *i.e.*, the temperature of the Pt-related CO peak. That hydrogen is, as discussed for the 0.9 ML as-prepared film, probably contributed by sites at the boundary of SmO*_x_* islands, *i.e.*, the SmO*_x_*-Pt interface at the surface. For the reduced film, the intensity of the H_2_ peak at 400 K is larger than that of the Pt-related H_2_ peak at 300 K which indicates that while the area of exposed Pt surface decreased during thermal reduction, the total length of the SmO*_x_* island boundaries increased. This interpretation is consistent with the wetting of a reduced SmO*_x_* film on Pt(111) observed in our previous STM work [13]. Over repeated TPD runs, the H_2_ peak that we relate to the SmO*_x_* island boundaries remains at a temperature of 400 K which suggests that the reaction and spillover mechanisms at the SmO*_x_*-Pt boundary on the surface do not change by the MeOH reaction and its effects on structure. The maxima of the SmO*_x_*-related CO and H_2_ desorption peaks are observed at an identical temperature of 600 K in the 1st TPD experiment with the reduced 0.9 ML Sm_2_O_3_/Pt(111) sample which is higher than that observed on the as-prepared film. The same trend was observed when as-prepared and reduced continuous SmO*_x_* films were compared. As discussed before, thermal reduction creates new adsorption sites for methoxy (*v*(C-O) band at 1005 cm^−1^ in IRRAS) on the SmO*_x_* surface which may cause a stronger binding between the methoxy species and SmO*_x_* and, thereby, higher desorption temperatures of the decomposition products. 

Over repeated TPD runs, the SmO*_x_*-related peaks shift to lower temperatures, e.g., the CO peak shifts from 600 K in the 1st TPD trace to 560 K in the 4th TPD trace. The average [H_2_]/{[CO]+[CO_2_]} ratio from 2nd to the 4th TPD is 2.38, *i.e.*, significantly larger than 2, the value expected from stoichiometry. The yield calculation suggests that 0.04 MLE C and O are missing in each TPD run, *i.e.*, 0.04 MLE oxygen remains on the reduced film and may slightly reoxidize it while 0.04 MLE carbon are also deposited on the surface. Slight reoxidation of the SmO*_x_* surface should reduce the number of surface defects/oxygen vacancies and can therefore explain the observed shift of SmO*_x_*-related TPD peaks to lower temperature upon repeated experiments. In the fourth TPD experiment, both SmO*_x_*-related CO and H_2_ peaks have shifted to about 550 K which is close to the temperature observed on the as-prepared 0.9 ML Sm_2_O_3_/Pt(111) sample in the sixth TPD experiment. Apparently, both the as-prepared and the reduced 0.9 ML SmO*_x_* films approach the same equilibrium condition during repeated MeOH TPD experiments. 

Structural information on the reduced 0.9 ML SmO*_x_*/Pt(111) sample was obtained using LEED, as shown in Figure 7b. Before the TPD experiments, a complicated satellite structure is observed which has been published in our previous work [13]. The satellite structure may be due to the coexistence of Sm_2_O_3_ (111) and SmO (100) domains and the formation of their coincidence lattice. After the TPD experiments, the LEED pattern exhibits a clear “rotated” structure which could be due to the coexistence of SmO*_x_* domains with different rotational mismatches between SmO*_x_* (111) trilayers and the Pt substrate. If there are multiple rotation angles, it is expected that the quasi-3 × 3 spots (which are due to double diffractions) are elongated because they are involved in different scattering vectors. Ritter *et al.*, have observed a similar rotated LEED pattern due to rotational mismatches between FeO (111) layers and a Pt(111) substrate [43]. Therefore, we assume that rotational mismatches between SmO*_x_* domains and the Pt(111) substrate are also responsible for the observed pattern. Moreover, the disappearance of the satellite structure indicates that SmO (100) vanishes after MeOH partially oxidizes the film. 

**Figure 8:**
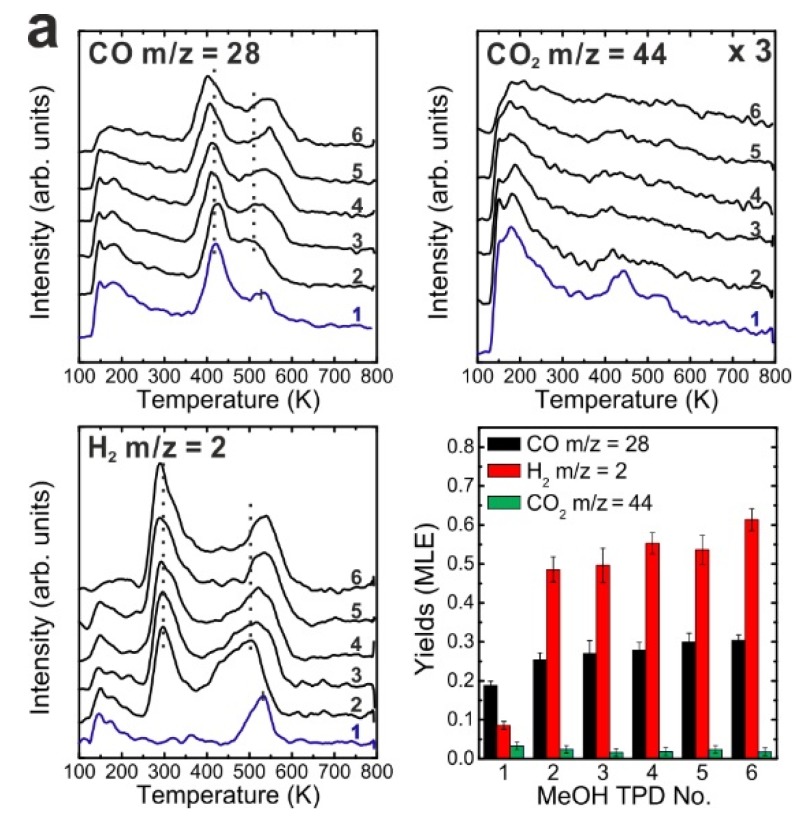

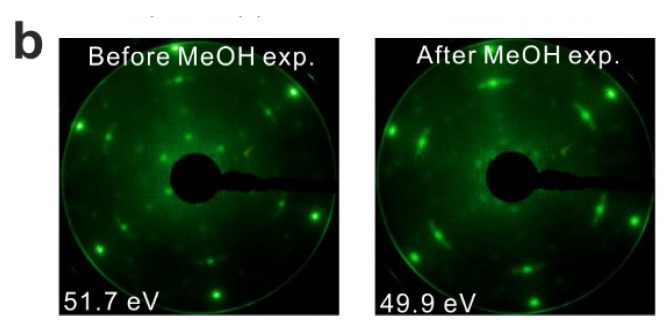
(**a**) Repeated TPD series collected on a 0.9 ML SmO*_x_*/Pt(111) surface after the treatment of reoxidation and the estimated yields of products, and (**b**) LEED results before and after the repeated MeOH TPD experiments.

A reoxidation treatment of the reduced 0.9 ML SmO*_x_*/Pt(111) sample was carried out by annealing in O_2_ (P = 5 × 10^−7^ mbar) at 1000 K for 10 min. (XPS spectra of the reoxidized sample are shown in Figure S2). The results of repeated MeOH TPD experiments performed on the reoxidized film are shown in Figure 8a. In contrast to our observations for the continuous SmO*_x_* film, reoxidation of reduced SmO*_x_* islands on Pt(111) leads to MeOH TPD spectra that are very similar to those observed for the as-prepared 0.9 ML Sm_2_O_3_/Pt(111) sample, *i.e.*, the chemical behavior of the as-prepared film is completely restored. This also holds for repeated TPD experiments. The result indicates that the reoxidation procedure (exposure of a film surface to molecular oxygen at elevated temperatures) is more efficient for a film surface than for bulk sites in the film. Therefore the 0.9 ML thin film, wetting the Pt surface after reduction and exhibiting only surface sites, would be restored more easily than the thicker (2.8 ML) reduced film. The Pt-related H_2_ and CO peaks have the same intensity as observed before the reduction-reoxidation cycle, indicating that the reoxidized Sm_2_O_3_ thin film dewets the Pt(111) substrate after the treatment, which is consistent with our finding in the previous STM work [13]. In that work, we demonstrated that the Sm_2_O_3_ (111) crystal structure is completely restored by the reoxidation treatment. However, in contrast to TPD, the LEED results obtained from the 0.9 ML reoxidized sample (Figure 8b) slightly differ from those obtained for an as-prepared film. Before the TPD experiments, there is a quasi-3 × 3 LEED pattern and two additional spots adjacent to the Sm_2_O_3_ (111) main spots. The additional spots may be caused by differently reoxidized Sm_2_O_3_ domains rotated by small angles or to surface areas that are still reduced, indicating that the film is not completely restored to its initial state. After the MeOH TPD experiments, the two additional spots adjacent to the Sm_2_O_3_ (111) main spots still remain and dim (2√3 × 2√3)R30° spots appear, obviously due to structural changes induced by the MeOH reaction. To resolve the origin of the (2√3 × 2√3)R30° structure, also observed on the 2.8 ML reduced film, further investigation is required. To sum up the results obtained upon reducing and reoxidizing the 0.9 ML SmO*_x_*/Pt(111) system, we found that the reduced film can be partially oxidized by MeOH during the repeated TPD runs and that, judging by the MeOH TPD experiments, the chemical behavior of an as-prepared 0.9 ML Sm_2_O_3_ film on Pt(111) is restored to the initial condition by a cycle of reduction and reoxidation treatments.

### 3.6. Discussion

Our results demonstrate that SmO*_x_* thin films exhibit a very high activity toward MeOH decomposition. We also find that MeOH decomposition on SmO*_x_* thin films induces structural changes in the film that modify the film's chemical reactivity towards MeOH, and *vice versa*. There are several studies showing how the structural properties of ceria can affect the surface reactivity toward MeOH. For instance, the observation that a reduced CeO*_x_*(111) surface exhibits higher reactivity than a fully oxidized CeO_2_(111) surface [18,19] was ascribed to the different coordination environment of surface Ce cations and O anions on these two surfaces. On the fully oxidized CeO_2_(111) surface, each top-layer O atom is coordinated to three Ce cations. Therefore, under-coordinated sites required for dehydrogenating MeOH [18,19] are lacking on this surface resulting in minor reactivity. Reduction of the CeO_2_(111) surface generates defect sites (O vacancies) which are responsible to the methoxy adsorption [44,45]. In accordance, the reduced CeO*_x_*(111) surface shows a higher reactivity than does the CeO_2_(111) surface. In contrast to Sm_2_O_3_, CeO_2_ exhibits a fluorite structure with both Ce and O ions adopting a hexagonal lattice within the (111) surface. Surface vacancies can be generated by reducing CeO_2_(111) to CeO*_x_*(111) (1.5 < *x* < 2), and different defect structures such as, e.g., a triangular superlattice of surface and subsurface oxygen vacancies have been demonstrated by SPM [44,45,46]. We have learned from our previous STM work that a Sm_2_O_3_ thin film grows on Pt(111) in a defective fluorite structure but does not form the bixbyite structure prevalent in bulk Sm_2_O_3_ [13]. The Sm cations at the surface are located in a hexagonal lattice whereas O anions are randomly distributed within a hexagonal lattice, resulting in plentiful vacancies/defects on the surface. Therefore, and since we have also observed a defect structure with a triangular superlattice in previous STM work, we consider the reaction of MeOH at the fluorite-related Sm_2_O_3_(111) thin film and a reduced CeO*_x_*(111) surface as comparable, and interpret the high activity of the SmO*_x_* thin film surface for MeOH dehydrogenation as due to the existence of abundant vacancies/defects on the surface.

A summary of our LEED results from the SmO*_x_* films on Pt(111) which provide evidence for even long-range structural changes within the films induced by MeOH is provided in the Figure S3 and Table S1. These structural changes are most likely connected to the MeOH capability of reducing or oxidizing SmO*_x_* thin films, depending on their initial oxidation state. *Vice versa*, the surface structure of the SmO*_x_* thin films does affect their chemical behavior and the reaction of MeOH on these films. Our IRRAS data reveal that on a reduced SmO*_x_* surface new adsorption sites can exist which are not observed on an oxidized surface. As a result, methoxy species can bind on these sites strongly and remain on the reduced surface up to higher temperatures than those on the oxidized surface.

**Figure 9:**
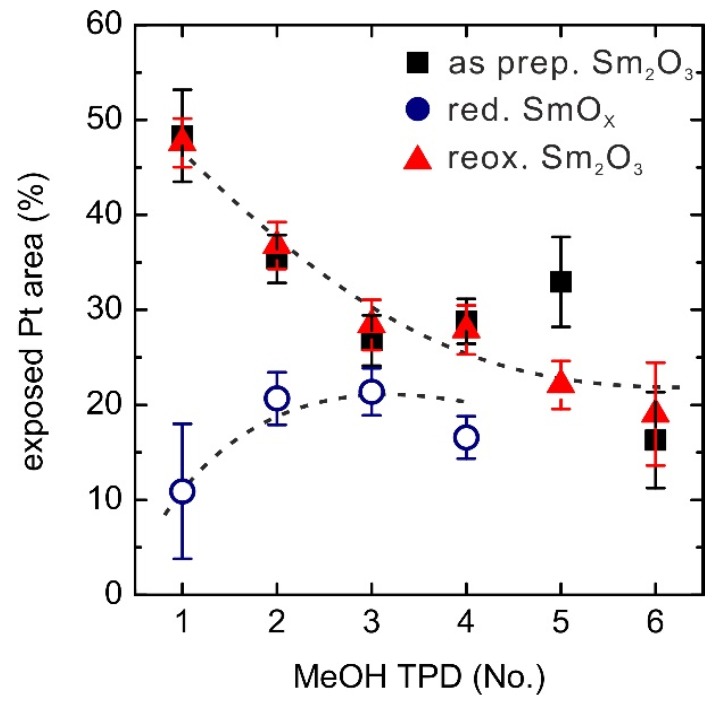
Changes of exposed Pt areas (%) on the 0.9 ML SmO*_x_*/Pt(111) samples over repeated TPD experiments. The dashed lines are added as a guide to the eye.

Apart from indications for changed oxygen vacancy densities at the surface induced by MeOH, our experiments on films of SmO*_x_* islands on Pt(111) also provide evidence for significant material transport involved in the structural changes related to the film's wetting/dewetting behavior which is demonstrated by our TPD results. (It may be interesting to note that wetting/dewetting induced by structural changes of the oxide was also observed for gold on ceria [47], which, inversely, is a metal-on-rare earth oxide system). Since the molecular desorption of MeOH is mainly contributed by the Pt(111) surface, the MeOH desorption yield during a TPD experiment is a good measure of the exposed Pt surface area, in particular for the 0.9 ML SmO*_x_*/Pt(111) samples. Akhter and White calculated the saturation monolayer coverage of MeOH and the maximum amount of reacted MeOH on a Pt(111) surface from their TPD experiments as 0.36 and 0.047 MLE, respectively [20]. Our calibration yields 0.04 ± 0.01 MLE MeOH reacting on the Pt(111) surface and 0.32 MLE intact MeOH desorbing from the Pt(111) surface which corresponds to the MeOH monolayer peak at 180 K. Following the same method, we determined that 0.08 ± 0.02 MLE of intact MeOH desorbs from the 2.8 ML Sm_2_O_3_ surface which is expected to exhibit no exposed Pt surface. Hence, we use a linear combination of the ratios of intact MeOH yield to total MeOH yield observed for bare Pt(111) and bare Sm_2_O_3_ (111) to estimate the exposed Pt area (%) on the 0.9 ML SmO*_x_*/Pt(111) samples as shown in Figure 9. About half of the Pt(111) surface is covered by the 0.9 ML as-prepared Sm_2_O_3_ thin film before the first TPD measurement which indicates that the film consists almost entirely of two-trilayer thick islands (one trilayer denoting an O-Sm-O layer). During repeated MeOH TPD runs, the exposed Pt area continuously decreases to ~20% of the surface by spreading of the SmO*_x_* over the Pt(111) surface. An 80% surface coverage with 0.9 ML SmO*_x_* can be only obtained when the film structure changes from two-trilayer high islands to almost entirely one-trilayer high islands.

The reduced SmO*_x_* film only exhibits ~10% exposed Pt surface before the first TPD experiment, meaning that ~90% Pt(111) surface is covered by the reduced SmO*_x_* thin film. As discovered in our previous STM work, a fraction of the Sm_2_O_3_ reduces to SmO at the interface between Pt(111) and Sm_2_O_3_ (111) by thermal reduction. The upper Sm_2_O_3_ layer spreads onto the Pt areas, as a result, so that finally about 90% Pt(111) surface is covered by a single trilayer of SmO*_x_*, consisting of Sm_2_O_3_ and SmO patches. During the repeated MeOH TPD runs, the exposed Pt area continuously increases from ~10% to ~20% suggesting a dewetting of the substrate by the film. Monitoring the changes of Pt-related CO and H_2_ peak intensities over the repeated TPD experiments confirms an increase of the exposed Pt area. Both Pt-related H_2_ and CO peaks are barely seen in the first TPD, indicating that the Pt surface is almost entirely covered by SmO*_x_*, but continuously increase during the repeated TPD runs. This result supports the interpretation that MeOH is capable of slightly reoxidizing the reduced 0.9 ML SmO*_x_* film. Furthermore, the reoxidation by molecular oxygen at elevated temperatures leads to further dewetting and finally exposes 50% of the Pt(111) surface. For repeated MeOH TPD runs after reoxidation, the change of the exposed Pt surface area shows the same trend as for the as-prepared Sm_2_O_3_/Pt(111) film, indicating that the sample condition is restored by the reoxidation process. It should be noted that the as-prepared and the reoxidized Sm_2_O_3_ films as well as the reduced films reach a similar condition with ~20% exposed Pt surface after repeated MeOH TPD experiments. The implication is that there is a single equilibrium condition of the SmO*_x_* film structure upon continuous exposure to MeOH, independent of the initial film condition.

A comparison of the overall activity observed for the MeOH reaction on clean Pt(111), the continuous 2.8 ML SmO*_x_* thin films, and the discontinuous 0.9 ML SmO*_x_* film is provided in Table 1. From the calibration of CO, CO_2_, and H_2_ yields, we estimated how many MLE of MeOH reacted on these surfaces. The overall activity follows the trend SmO*_x_*/Pt(111) > SmO*_x_* >> Pt(111) and suggests that a cooperative-effect between SmO*_x_* and Pt on the 0.9 ML SmO*_x_*/Pt(111) samples must play an essential role for promoting the decomposition of MeOH. A likely explanation of this observation is a spillover of CO and H from SmO*_x_* islands to exposed Pt surface occurring at low temperature (~250 K). As a result, adsorption sites (on-top sites) on SmO*_x_* can be vacated. As the tail of the MeOH monolayer-desorption feature in TPD up to 250 K indicates, there is still molecular MeOH available on the surface which can fill the vacant methoxy adsorption sites on the SmO*_x_* surface. Therefore, in total, more methoxy can react on the 0.9 ML SmO*_x_*/Pt(111) samples than on the continuous 2.8 ML SmO*_x_* thin films. For a continuously driven reactor these SmO*_x_* adsorption sites (on-top sites) in close proximity to Pt(111) areas should be relevant as they sustain cycles of methoxy decomposition—spillover of the products—readsorption of methoxy at relatively low temperature.

**Table 1:** Summary of the overall reactivity for the MeOH reaction on Pt(111), the 0.9 ML SmO*_x_*/Pt(111), and the 2.8 ML SmO*_x_* samples. Yields of methanol reaction and ratios of [H_2_]/{[CO] + [CO_2_]} estimated from TPD spectra on each samples. Yields of methanol reaction are defined by [CH_3_OH] = [CO] + [CO_2_] except on the 0.9 ML reduced film (marked *), 0.35 MLE is estimated by [CH_3_OH] = 2 [H_2_] because a fraction of carbon and oxygen remains on/in the reduced film.

TPD Spectra	Pt(111)	0.9 ML Samaria Thin Films			2.8 ML Samaria Thin Films		
	--	as prep. Sm_2_O_3_	red. SmO_x_	reox. Sm_2_O_3_	as prep. Sm_2_O_3_	red. SmO_x_	reox. Sm_2_O_3_
**Yield of MeOH reaction (MLE)**							
1st TPD	0.04	0.24	0.26	0.22	0.27	0.12	0.14
Ave. w/o 1st TPD	--	0.31	0.35 *	0.30	0.21	0.17	0.14
**Ratio of [H_2_]/{[CO]+[CO_2_]}**							
1st TPD	1.80	0.89	2.00	0.40	--	0.57	0.22
Ave. w/o 1st TPD	--	1.87	2.38	1.80	1.61	1.59	1.35

## 4. Conclusions

**Figure 10:**
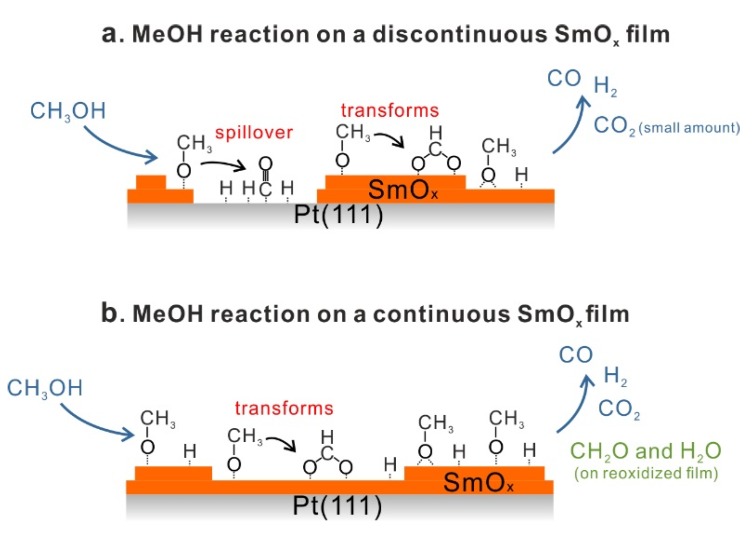
Summary of the MeOH reaction on (**a**) a discontinuous SmO*_x_* thin film, and (**b**) a continuous SmO*_x_* thin film.

We investigated the adsorption and reaction of MeOH on both continuous and discontinuous SmO*_x_* thin films on Pt(111) by TPD and IRRAS. Combining the available results, we conclude that MeOH is dehydrogenated forming mainly CO and H_2_ as desorbing products. On as-prepared continuous and discontinuous Sm_2_O_3_/Pt(111) samples, CO_2_ is also produced in small quantities via CO oxidation by oxygen from the Sm_2_O_3_ surface. According to the yield estimation of products, we observe the highest reactivity for the discontinuous SmO*_x_* film. The product yields obtained from the discontinuous film are, on average, a factor of 1.6 higher than those obtained from the continuous film. This observation can be explained by a spillover of products from SmO*_x_* islands to exposed Pt areas, a mechanism which is not available on continuous films. All identified MeOH reaction mechanisms are summarized in Figure 10, while all detected IRRAS vibrational frequencies and their assignments are summarized in Table 2.

**Table 2:** Vibrational frequencies and assignments of methanol, methoxy and formate species collected from the 0.7 ML and 2.3 ML SmO*_x_*/Pt(111) samples. The corresponding IRRAS spectra of the 2.3 (± 0.2) ML thick continuous SmO*_x_* film are shown in Figure S4.

Assignments	Vibrational Frequencies						
	Methanol	Methoxy and Formate Species					
	(cm^−1^)	0.7 ML Sm_2_O_3_ (cm^−1^)	0.7 ML red. SmO*_x_* (cm^−1^)	0.7 ML reox. Sm_2_O_3_ (cm^−1^)	2.3 ML Sm_2_O_3_ (cm^−1^)	2.3 ML red. SmO*_x_*(cm^−1^)	2.3 ML reox. Sm_2_O_3_ (cm^−1^)
*ν*(C-O)	1046	1080–1077	1089–1077	1084–1078	1083–1074	1065–1057	1080–1072
		1065	1061	1055–1052	1062	1015	1066
		1042	1037		1049		1050
			1005				
*ρ*(CH_3_)	1130	1140	1140				
*ν_s_*(OCO), *δ*(CH)		1378	1378	1377	1377		1378
*ν*(C≡O) ^a^		~2080	~2080	~2088	~2089		
*ν_s_*(CH_3_)	2831	2790	2790	2780	2780	2800	2780
2*δ*(CH_3_), *ν*(CH)	*	2856	2851	2850	2840	2854	2837
*ν_as_*(CH_3_)	2957	2926	2926	2941	2925	2938	2920
*ν*(OH)	3280						

Notes: ^a^ The band corresponds to carbon monoxide on the exposed Pt(111) surface. * The overtone band in case of methanol sits underneath the CH_3_ symmetric and asymmetric stretch bands.

Effects of the SmO*_x_* structure on the MeOH reaction, and *vice versa*, were revealed by studying as-prepared, reduced, and re-oxidized films. The highest reactivity was observed for a discontinuous SmO*_x_* film after thermal reduction, containing an unknown amount of SmO domains. Moreover, the MeOH decomposition leaves some amount of oxygen on the reduced film, indicating surface reoxidation by MeOH. The SmO domains most likely promote reduction reactions while being oxidized. However, one may conceive that the phase boundaries should also play a dominant role for the overall reactivity of the film. Therefore, while we find evidence, e.g., for new adsorption sites for methoxy on the reduced film, it cannot unambiguously assigned to the SmO(100) phase. CO_2_ is detected from both continuous and discontinuous films, but the highest CO_2_ yield was observed for the as-prepared continuous film suggesting that there is an oxygen species available for oxidation reactions on SmO*_x_* films, which is not necessarily expected from a rare earth sesquioxide. Apart from the effect of the film’s surface structures on the MeOH reactivity, the decomposition of MeOH itself also modifies the SmO*_x_* structure as revealed by repeated TPD experiments. They probably involve oxygen vacancy formation at the surface.

Our results indicate that whether as-prepared or reoxidized or reduced discontinuous films are used as a starting point, MeOH decomposition on these films drives the SmO*_x_* toward a structural equilibrium condition. The SmO*_x_*-related desorption signals of products obtained for both oxidized and reduced films over repeated TPD runs reach a similar temperature of maximum desorption (~550 K) while the fraction of exposed Pt area adjusts to ~20% of the entire surface area. Finally, the repeated TPD experiments reveal that hydrogen is stored either in the film or as OH groups on the SmO*_x_* surface. Further investigations are in progress to elucidate the origin of the capability of SmO*_x_* films for hydrogen storage.

## Supplementary Materials

Supplementary materials can be accessed at: http://www.mdpi.com/1996-1944/8/9/6228/s1.
