# Supplementary material for: Methanol Adsorption and Reaction on Samaria Thin Films on Pt(111)
Source: Materials (Basel). 2015 Sep 17;8(9):6228–56. doi: 10.3390/ma8095302 (PMC5512909; doi:10.3390/ma8095302)
Supplement: Supplementary file 1 [file materials-08-05302-s001.pdf]

Supplementary Information

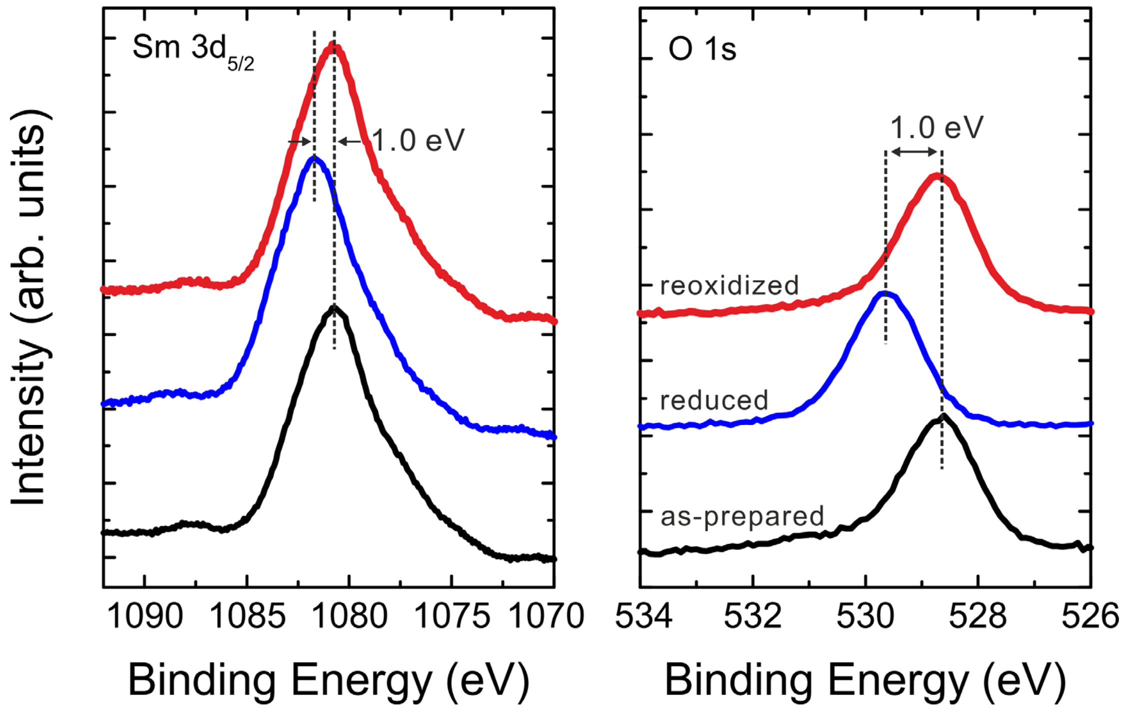

Figure S1. XPS spectra of Sm $3d_{5/2}$ and O 1s regions collected from the 2.8 ML as-prepared, reduced, and reoxidized SmO_x thin films.

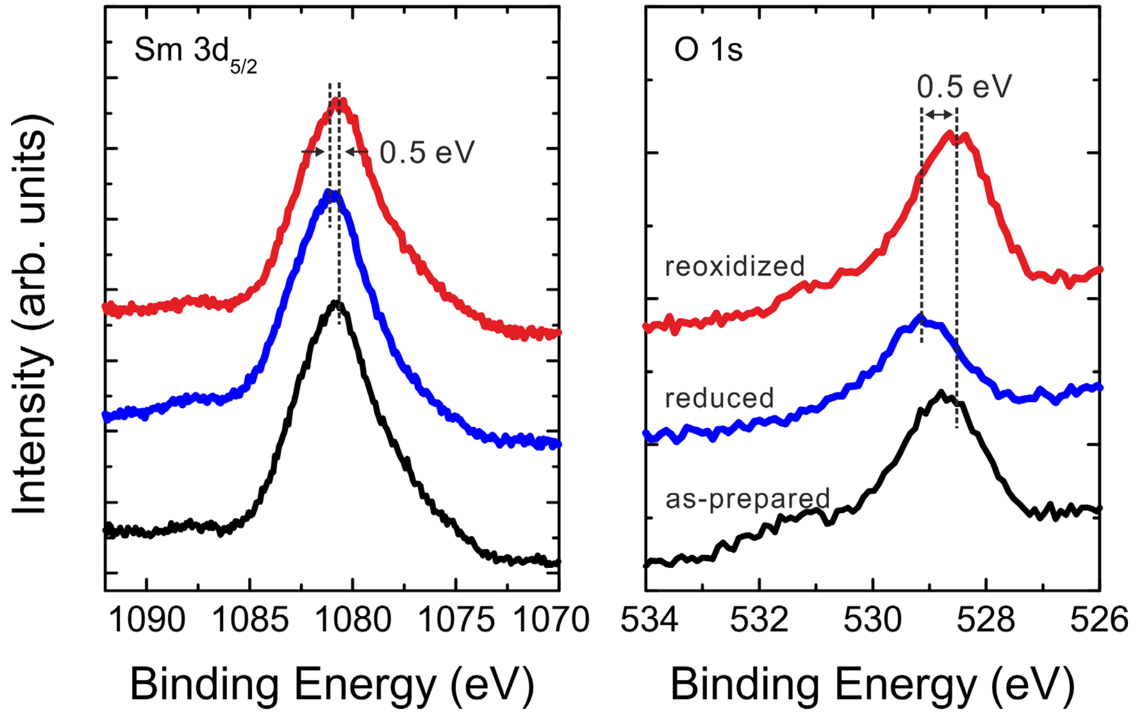

Figure S2. XPS spectra of Sm $3d_{5/2}$ and O 1s regions collected from the 0.9 ML as-prepared, reduced, and reoxidized SmO_x thin films.

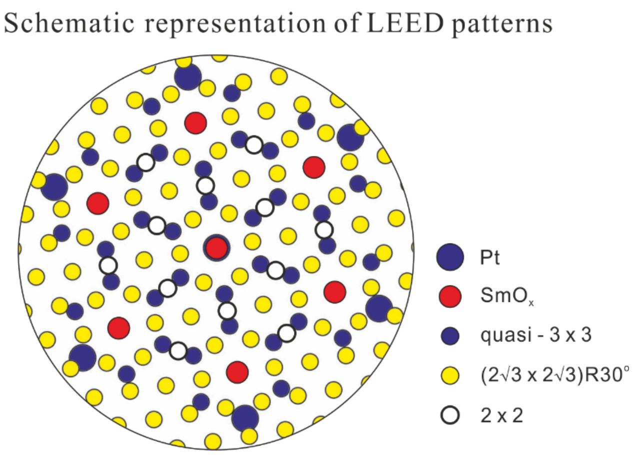

Figure S3. Summary of LEED patterns observed.

Table S1. Summary of LEED patterns observed for the differently prepared SmO_x deposits.

MeOH TPD Treatment	LEED Patterns of 0.9 ML SmO_x on Pt(111)		
	**as-prepared Sm_2O_3**	**reduced SmO_x**	**reoxidized Sm_2O_3**
w/o	quasi-3 × 3	satellites	quasi-3 × 3 + 2 add. spots
with	slightly rotated	rotated	quasi-3 × 3 + 2 add. spots + (2√3 × 2√3)R30°
**MeOH TPD Treatment**	**LEED Patterns of 2.8 ML SmO_x on Pt(111)**		
	**as-prepared Sm_2O_3**	**reduced SmO_x**	**reoxidized Sm_2O_3**
w/o	quasi-3 × 3	quasi-3 × 3 + (2√3 × 2√3)R30°	quasi-3 × 3 + 2 × 2
with	SmO_x(1 × 1)	quasi-3 × 3 + (2√3 × 2√3)R30°	quasi-3 × 3 + (2√3 × 2√3)R30°

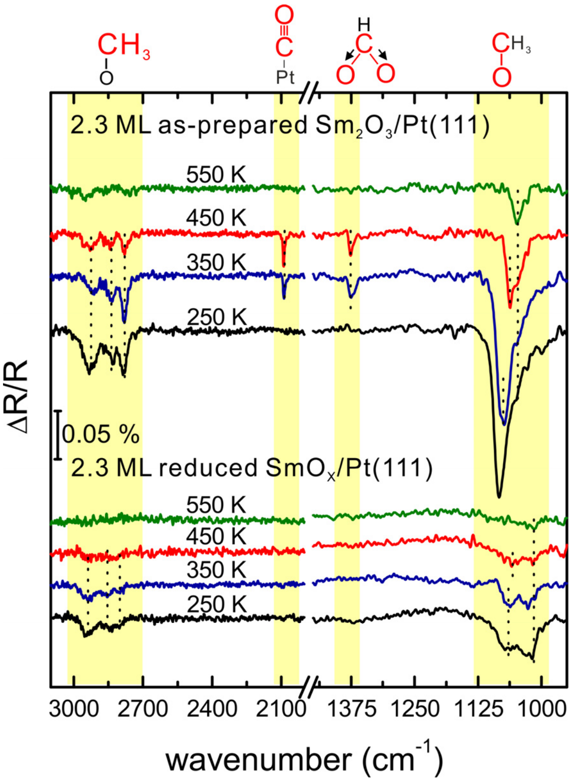

Figure S4. Temperature-dependent IRRAS spectra collected from the 2.3 ML as-prepared and reduced SmO_x thin films.
